# The Preparation of the Lying-in Patient and the Management of Normal Labor*Read at Waco meeting, Central Texas Medical Association, July 10, 1900.

**Published:** 1900-12

**Authors:** J. H. Alexander

**Affiliations:** Meridian, Texas


For Texas Medical Journal.

### The Preparation of the Lying-in Patient and the Management of Normal Labor.*

BY J. H. ALEXANDER, M. D., MERIDIAN, TEXAS.

The real preparation of the lying-in patient should begin as early in gestation as possible, and continue to the time of labor, whether normal or abnormal.

The obstetrician's duty to his patient can not be properly discharged unless he can have the patient under his care as early as the sixth or seventh month. The first step in the preparation of the lying-in patient should be to prepare her mind by the method of suggestion to a thorough knowledge of the physiological process of bearing children, and suggest the probable result of proper hygienic care and living on herself and offspring.

By the terms suggested I mean it as near in its strict sense as is within the judgment of the physician necessary to produce the result desired. By this means you can gain the confidence of the patient, and can thereby have her follow your directions very closely, and this, if properly given, is half the battle. As to the habits and diet of the patient it is a very important matter to both mother and child, as well as to the physician who is to be the accoucheur. First, you should suggest to the patient the proper hours to work or exercise and rest. Exercise should be very moderate and taken three or four times during the day, in the open air if possible; rest should be indulged in freely and especially plenty of good, refreshing sleep. Rest in the daytime should be in the recumbent posture three or four hours during the day, with eight or ten hours for sleep at night. If the patient be literary in her taste you should prescribe the class of her reading matter, and have none but the very purest kind. The next rule of hygiene should be the dieting of the patient.

This should consist of fresh and cooked fruits, cereals and plenty of sweet milk, or, if this can not be taken, use buttermilk instead; and especially is this last important if there should be albuminurea or great edema of limbs or body. And now we can greatly relieve the abdominal tension by gentle massage, using for the purpose of softening the tissues some soft ointment, lanolin, cocoa oil or good hog's lard nicely perfumed; making use of the massage twice daily,

*Read at Waco meeting, Central Texas Medical Association, July 10, 1900.

beginning as early as there is any abdominal tension; this wlil also prevent the formation of atrophic stria, and add greatly to the comfort of the patient in question, if proper suggestion is made as to the result of such procedure.

The next thing you should direct your patient about is the emptying of the lower bowel, which can very often be accomplished in a very satisfactory manner by suggestion and the regulation of habit about going to stool, or if necessary by small doses of some saline laxative taken before breakfast, such as sulph. mag. or, as I prefer, Abbott's Saline Laxative, which is more pleasant to take than salts. By due attention to the bowels we will relieve our patient of all the disagreeable sequelæ of constipation, piles, etc., and thereby add greatly to her comfort and to our own success in the management of normal labors.

We now have our patient to term and should proceed to prepare her for her lying-in proper. First, the patient should be instructed that upon the first symptoms of labor she should take an all over bath and go to bed in a bed prepared for her labor, between clean sheets. This should be done when the accoucheur is sent for, and upon his arrival he should ascertain if it has been done. If so, and labor has begun, he should cleanse his hands by any of the antiseptic methods (I use etherial antiseptic soap with nail brush, afterwards rinsing the hands in one to one thousand bichloride solution), after which he should give an antiseptic douche, cleansing the external parts as well, and then making a bimanual examination, at which he should be able to ascertain the diameter of the pelvis, the condition of the soft parts, and whether the presentation is head or breach, reserving diagnosis as to position until os is more fully dialated, which, presuming to be normal, O. L. A., we then have very little to do save cheer up patient and make her as comfortable as possible, using a little chloroform for this if necessary. We should now prepare for any emergency, and especially the necessity of a normal case. On a table conveniently situated we should have ligatures for cord, scissors, chloroform, inhaler, ergot or ergotol and clean towels. Forceps, needles and holders, silk worm gut or wire sutures should be in obstetrical bag.

The duties of the physician in the first stage of normal labor are very simple, first directing the patient how to use the pains to the greatest advantage, and make an occasional examination to ascertain the progress of this stage. When the os is very nearly dilated it will greatly facilitate matters if the os can be passed over the occiput, just after the membranes have ruptured, which should be done about this time if it does not occur spontaneously.

In the second stage the examination should be made more frequently to ascertain what degree of descent has been made, and in order to support the perineum at the proper time. When occiput begins to pass under and over the pubis, the finger should be introduced into the rectum and occiput raised by using the finger on the chin or in the mouth of the child, making as it were a fulcrum of the neck. Both hands should now be used to rotate the head and deliver the shoulders, which should be done by the hand and face sweeping process, and especially if the tension is very great on the perineum and the shoulders very large, as the perineum is in more danger from the shoulders than the head. The cord being now freed, if around the neck, the child should be made to breathe by the introduction of the finger in the mouth or any other of the very many other processes if this fail. After pulsation in the cord becomes very weak the cord should be tied and cut, and the baby turned over to a nurse or wrapped up in a warm shawl or blanket, and then direct your attention to the third stage.

Now, there is some dispute just here as to the use of ergot. I use it in some cases, but not in all, and I think that if we are going to give it at all it should be given after delivery of the child. There are more ways than one of delivering the secundines, by cord traction, Crede method, etc., with which you are too well acquainted to necessitate a description. I always use the following method: Place the left hand over the fundus uteri and pass finger of right hand along the left wall of the vagina until I reach the margin of the placenta, and with pressure from above I deliver precenta with the edge of margin down. I have never made a satisfactory delivery of the secundines by cord tractions. After delivery of secundines I continue to knead the uterus for ten or fifteen minutes, and then repeat every five or ten minutes until I get firm contractions, thereby expelling all clots and preventing troublesome after pains. The child is now taken in charge and the anus examined, cord dressed in soft gauze saturated in hog's lard, and the eyes thoroughly cleansed. This done, I have baby bathed, soiled clothing removed and napkin applied, after which I direct as to diet, kidneys and bowels. Having done all this I feel that my duty has been discharged to my patron, my fee well earned, and with a clear conscience I leave my patient and take a needed rest.

